# Secret lifestyles of *Neurospora crassa*

**DOI:** 10.1038/srep05135

**Published:** 2014-05-30

**Authors:** Hsiao-Che Kuo, Sun Hui, Jaeyoung Choi, Frederick O. Asiegbu, Jari P. T. Valkonen, Yong-Hwan Lee

**Affiliations:** 1 Department of Forest Sciences, University of Helsinki, Helsinki, Finland; 2 Department of Agricultural Sciences, University of Helsinki, Helsinki, Finland; 3 Department of Agricultural Biotechnology, Center for Fungal Pathogenesis, and Center for Fungal Genetic Resources, Seoul National University, Seoul 151-921, Korea

## Abstract

*Neurospora crassa* has a long history as an excellent model for genetic, cellular, and biochemical research. Although this fungus is known as a saprotroph, it normally appears on burned vegetations or trees after forest fires. However, due to a lack of experimental evidence, the nature of its association with living plants remains enigmatic. Here we report that Scots pine (*Pinus sylvestris*) is a host plant for *N. crassa*. The endophytic lifestyle of *N. crassa* was found in its interaction with Scots pine. Moreover, the fungus can switch to a pathogenic state when its balanced interaction with the host is disrupted. Our data reveal previously unknown lifestyles of *N. crassa*, which are likely controlled by both environmental and host factors. Switching among the endophytic, pathogenic, and saprotrophic lifestyles confers upon fungi phenotypic plasticity in adapting to changing environments and drives the evolution of fungi and associated plants.

The filamentous fungal species, *Neurospora crassa* has become a popular experimental model microbe for genetic, cellular, and biochemical research in the latter half of the 20^th^ century^1^,^2^,^3^. *N. crassa* is commonly found on carbohydrate-rich foodstuffs and residues of sugar-cane processing^1^. Most *N. crassa* strains collected for studies of geographical distribution have been from tropical and subtropical regions^4^. However, *N. crassa* and *N. discreta* can also be found as far north as Montana and Alaska, respectively^5^, a natural habitat that contains coniferous trees. Although the lifestyle of *N. crassa* in the wild is unknown, it normally appears on burned vegetations or trees after forest fires^5^,^6^. It has been suggested that heat from forest fires stimulates the germination of ascospores in the soil and provides a sterile, nutrient-containing environment that stimulates growth^5^. *Neurospora* species has also been isolated from the artificial plantation of *Acer ginnala* (Amur Maple) in northeastern China and proposed to be an endophyte^7^; however, no strong experimental evidence of its association with living plants is available. Global distribution and comprehensive collection of isolates of *Neurospora* species offer a new platform to decipher its ecology and evolution in nature^4^,^5^. Fungi have been traditionally categorized as parasites, symbionts, or saprotrophs based on their strategies for nutrient acquisition. However, accumulated evidence suggests that fungal lifestyles are plastic in relation to their hosts and environments, rather than rigidly dictated only by their genetic makeup^8^,^9^,^10^,^11^. Fungal endophytes exhibit a broad range of lifestyles (*e*.*g*., latent saprotrophs or pathogens, and mutualists), which are determined by the fitness benefits conferred on their hosts, the production of secondary metabolites, and/or their colonization strategies^8^,^12^,^13^,^14^. Mycorrhizal fungi can also act as endophytes, necrotrophic pathogens, and antagonists of host or non-host plants^8^,^9^. Here, we performed a series of experiments to reveal the alternative lifestyles of *N. crassa* and provide the first evidence of endophytic and pathogenic *N. crassa* lifestyles in Scots pine (*Pinus sylvestris*).

## Results

### Association of *N. crassa* with Scots pine

In order to investigate the alternative lifestyles of *N. crassa*, Scots pine seedlings grown in microcosm were inoculated with conidial suspension (10^5^ conidia/ml) and the colonization patterns were documented over a period of 5 months by fluorescence and confocal microscopy (Supplementary Fig. S1). Most of seedlings looked healthy and were indistinguishable from those without inoculation. Surprisingly, however, fungal hyphae expressing GFP were observed from inside of inoculated seedlings, but not from uninoculated ones. During its growth in the cells of Scots pine seedlings, *N. crassa* was found to proliferate and survive for up to 5 months without causing any disease symptoms, suggesting its endophytic lifestyle. Inside the roots, fungal growth was confined mostly to the root epidermis and cortex layers (Supplementary Fig. S1e). More compelling evidence of endophytic lifestyle is being described in the later section. To further decipher the innate association between *N. crassa* and Scots pine, we performed a series of inoculation experiments on Scots pine seedlings grown on water agar.

### Can *N. crassa* be a plant pathogen on Scots pine?

**Figure 1:**
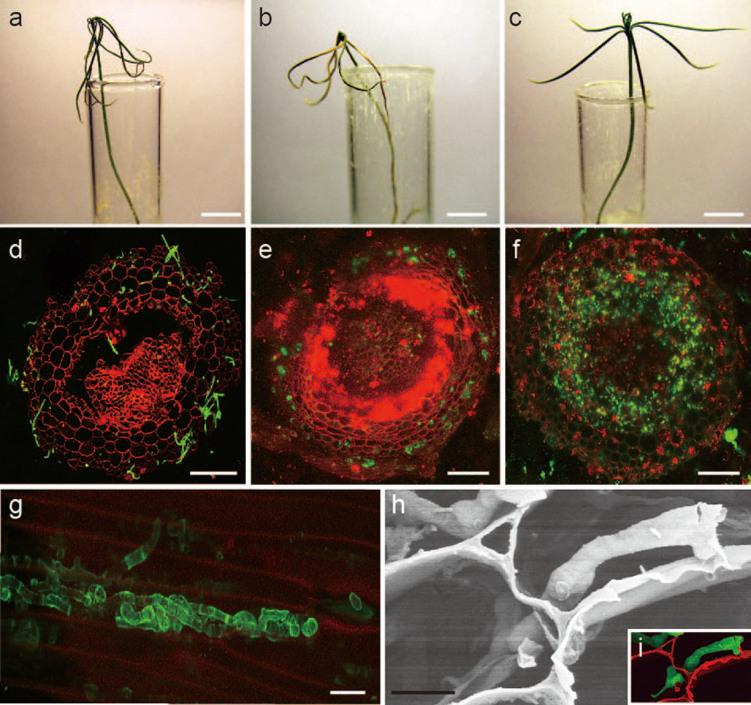
Pathogenic interactions between *N. crassa* and Scots pine seedlings. Scots pine seedlings were inoculated with *N. crassa* (**a**), *H. annosum* (**b**), and control (**c**). (**d**) Transverse section of Scots pine root inoculated with *N. crassa*. Plant cell walls were stained with PI, and fungal hyphae were labeled with WGA. (e and f) Transverse sections of Scots pine roots inoculated with *N. crassa* FGSC 10589. GFP images were obtained by staining with FM4-64 at different stages of infection from 3 (**e**) to 5 (**f**) weeks post inoculation (wpi). (**g**) Image of *N. crassa* hyphae stained with WGA within host plant cells. (**h**) SEM image of *N. crassa* hyphae growing from one plant cell to another. (**i**) Colored SEM image, red and green indicate plant cell wall and *N. crassa* hyphae, respectively. Bars = 1 cm (**a**–**c**); 100 μm (**d**, **e**, and **f**); 10 μm (**g**); 5 μm (**h**). *N. crassa* strains used in **a**, **d**, **g**, **h**, and **i** was FGSC 2489.

**Figure 2:**
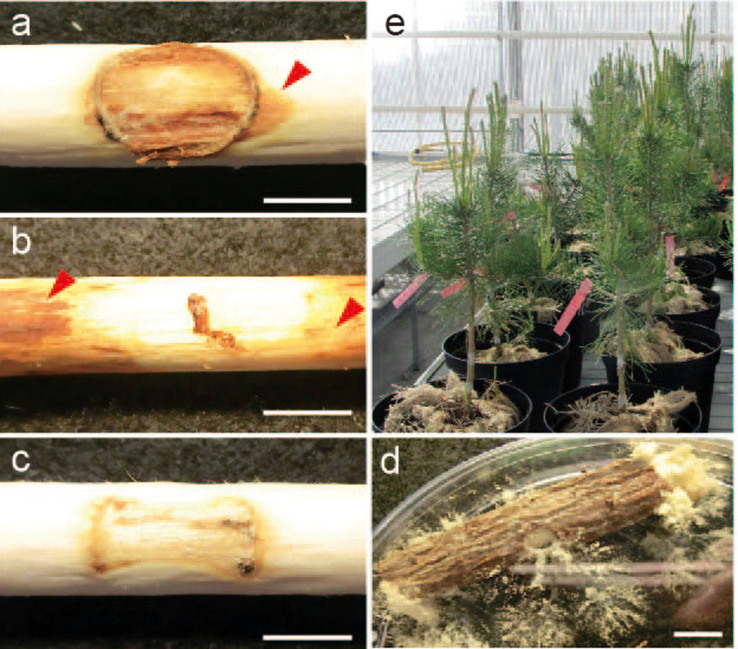
Infection of 3-year-old Scots pine trees by *N. crassa*. Disease symptoms caused by *N. crassa* (**a**), *H. annosum* (**b**), and control (**c**). Disease symptom was measured 6 wpi. Red arrows indicate typical necrosis symptoms. (**d**) Survival of *N. crassa* in plant tissues after heat treatment. Three-year-old *N. crassa*-infected trees were heat-treated to 121°C at 100 kPa for 10 min, followed by incubation at 24°C for 2 weeks. (**e**) Three-year-old Scots pines used for infection experiments in the greenhouse. Bar = 1 cm.

Our most remarkable finding is that *N. crassa* can act not only as an endophyte but also as a pathogen on Scots pine, when the host plant was grown on water agar or under controlled environments in the greenhouse. Infection with *N. crassa* incited typical disease symptoms, eventually causing the death of Scots pine seedlings. The mortality rate reached to 83% (90 out of 108 seedlings) at 5 weeks post inoculation (wpi) (Fig. 1a). The abiosis of Scots pine caused by *N. crassa* takes 4–5 weeks, whereas its well-adapted fungal pathogen *Heterobasidion annosum* exerts a similar effect in only 3–4 weeks (96% mortality rate) (Fig. 1b). During the initial stage of infection, *N. crassa* conidia germinated, formed a hyphopodia-like structure (Supplementary Fig. S2c), penetrated into plant tissues, and grew intra- or intercellularly between adjacent cells (Fig. 1d–i; Supplementary Fig. S2d–f; Supplementary Fig. S3). Invasive growth continued from root cortical cells (Fig 1d–e) to the core area (Fig. 1f), and they were found in almost 50% of infected root cells at 5 wpi (Supplementary Fig. S4). At the end of the infection stage, *N. crassa* hyphae could grow out from the stomata on the stem of infected Scots pine seedling (Supplementary Fig. S5a–f) and develop conidiophores with conidia (Supplementary Fig. S5g). These observations clearly demonstrate that *N. crassa* can complete its life cycle in association with Scots pine and further support the hypothesis that *N. crassa* has a pathogenic lifestyle. Moreover, culture filtrate of *N. crassa* induced similar cell death in Scots pine seedlings (Supplementary Fig. S6b), suggesting that *N. crassa* may produce phytotoxic compounds and function as a necrotrophic plant pathogen on Scots pine. To understand if *N. crass* can incite disease symptom not only in seedlings grown on water agar, but also grown trees, 3-year-old Scots pine trees were inoculated with wood dowels pre-colonized by *N. crassa* in the greenhouse. *N. crassa* could incite clear necrosis on 3-year-old trees 6 wpi. The necrosis areas by *N. crassa* and *H. annosum* were 42.5% and 67.2%, respectively, when measured by the ratio of the white (healthy) and brown area (necrosis) (Fig. 2a–c). These infected trees were sampled and heat-treated at 121°C for 10 min to understand whether *N. crassa* inside of tree could survive under harsh conditions such as forest fire. Surprisingly, *N. crassa* was grown out and was the sole surviving fungal taxon on wood trunks after heat treatment when incubated for 2 weeks (Fig. 2d). This further supports our previous finding and suggests that *N. crassa* within host cells can survive as a pathogen or an endophyte and grow out from the burned tree as a saprotroph.

### Biochemical and molecular mechanisms underlying *N. crassa* and Scots pine interactions

**Figure 3:**
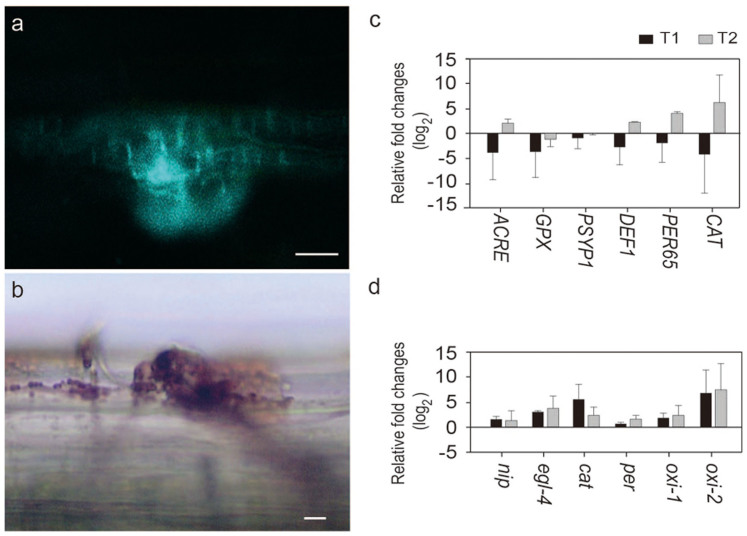
Host response and gene expression during interaction with *N. crassa*. (**a**) Callose deposition around the infection site on stem, stained with aniline fluorochrome. (**b**) Accumulation of reactive oxygen species (ROS) at the infection site on stem, stained with diaminobenzidine (DAB). Bars = 20 μm. (**c**) Expression profiles of defense-related genes in the roots of Scots pine after *N. crassa* inoculation: *ACRE*, Avr9/Cf-9 rapidly elicited defense-related gene; *PER65*, peroxidase 65; *PSYP1*, class III peroxidase; *GPX*, glutathione peroxidase; *DEF1*, defensin; and *CAT*, catalase. (**d**) Expression profiles of *N. crassa* genes during its interaction with Scots pine's roots. *nip*, Necrosis-inducing protein; *cat*, catalase-1; *per*, dyp-type peroxidase; *oxi-1* and *oxi*-2, two oxidoreductases. The level of expression was measured at one (T1) and two (T2) weeks after inoculation. Fold changes are relative to uninfected Scots pine seedlings (**c**) and *N. crassa* grown on Vogel's medium (**d**). The bars indicate standard deviation among 3 biological replicates.

To determine whether *N. crassa* infection elicits a defense response in Scots pine seedlings, host-defense-related reactions were observed. Plant cells at infection sites killed by *N. crassa* were observed by staining with Evans blue (Supplementary Fig. S6a). Furthermore, callose deposition and the accumulation of ROS around infection sites on stem were evident following staining with aniline fluorochrome blue (Fig. 3a) and diaminobenzidine (DAB) (Fig. 3b), respectively. These data indicate that the interaction between *N. crassa* and Scots pine represents a typical host–pathogen interaction.

In addition to biochemical response, expression patterns of ROS- and defense-related genes, such as those encoding peroxidases^15^ (peroxidase 65 [*PER65*], class III peroxidase [*PSYP1*], and glutathione peroxidase [*GPX*]), Avr9/Cf-9 rapidly elicited defense-related gene (*ACRE*)^16^, defensin (*DEF1*)^17^, and catalase (*CAT*)^15^ were monitored by qRT- PCR. All genes were differentially expressed in Scots pine's roots at 2 wpi with *N. crassa* (Fig. 3c). Expression of genes encoding catalase and peroxidases was most highly up-regulated. Expression of *ACRE* and *DEF1* was also up-regulated in the roots (Fig. 3c) and stems (Supplementary Fig. S7a)after *N. crassa* infection. We also monitored the expression profiles of pathogenicity-related genes in *N. crassa*, including those encoding necrosis-inducing protein (*nip*)^18^, endoglucanase IV (*egl-4*)^19^, catalase (*cat*), peroxidase (*per*), and two oxidoreductases (*oxi-1* and *oxi-2*).

Expression of *egl-4* was highly up-regulated in roots (Fig. 3d and Supplementary Fig. S7b). Similar expression pattern of *egl-4* was observed in *H. irregulare* during infection of Scots pine^19^. Expression of the genes encoding the two oxidoreductases and catalase were also highly up-regulated during interaction with Scots pine seedling (Fig. 3d). Together, expressions of genes responsible for ROS modulation were highly up-regulated in both the host and the pathogen. ROS is known to play key roles in maintaining the balance between endophytic and pathogenic fungal lifestyles with host plants^20^. Reducing ROS levels in the host can stimulate latent pathogens to cause disease^20^,^21^. Our gene expression data suggest that the pathogen and the host use opposite strategies to manipulate the level of ROS to maintain their relationships. Scots pine appears to reduce the expression of catalase (leading to accumulation of ROS) (Fig. 3c), resulting in a toxic response to the pathogen^22^ in the roots. In *N. crassa*, on the other hand, expression of catalase gene was highly up-regulated at an early stage of root infection (Fig. 3d), reducing the plant-derived ROS level. Later, the host catalase begins to be produced at high levels (Fig. 3c), likely due to excess ROS caused by the presence of the pathogen. Catalase expression of *N. crassa* was down-regulated, maintaining the virulence (Fig. 3d) of the fungus to the host, which can lead to an ectopic oxidative burst and cell death (accumulation of ROS)^23^. At the same time, both the pathogen and the host were preparing for the next phase of interactions in the other part of plant such as stem cells (Supplementary Fig. S7a,b). Since expression of a gene encoding a flavoprotein oxidoreductase (NCU06061)^24^ was highly up-regulated during infection (Fig. 3d), two deletion mutants (Δ*oxi-1* and Δ*oxi-2*) were tested for their virulence. Indeed, deletion mutants showed significantly lower virulence on Scots pine seedlings (*p* = 0.02 and 0.07 in Δ*oxi-1* and Δ*oxi-2*, respectively) (Supplementary Fig. S8), although they did not have any defect on mycological characters including mycelial growth, colony morphology and pigmentation. These combined biochemical and gene expression data suggest that the association between *N. crassa* and Scots pine is a typical intimate host–pathogen interaction.

## Discussion

**Figure 4:**
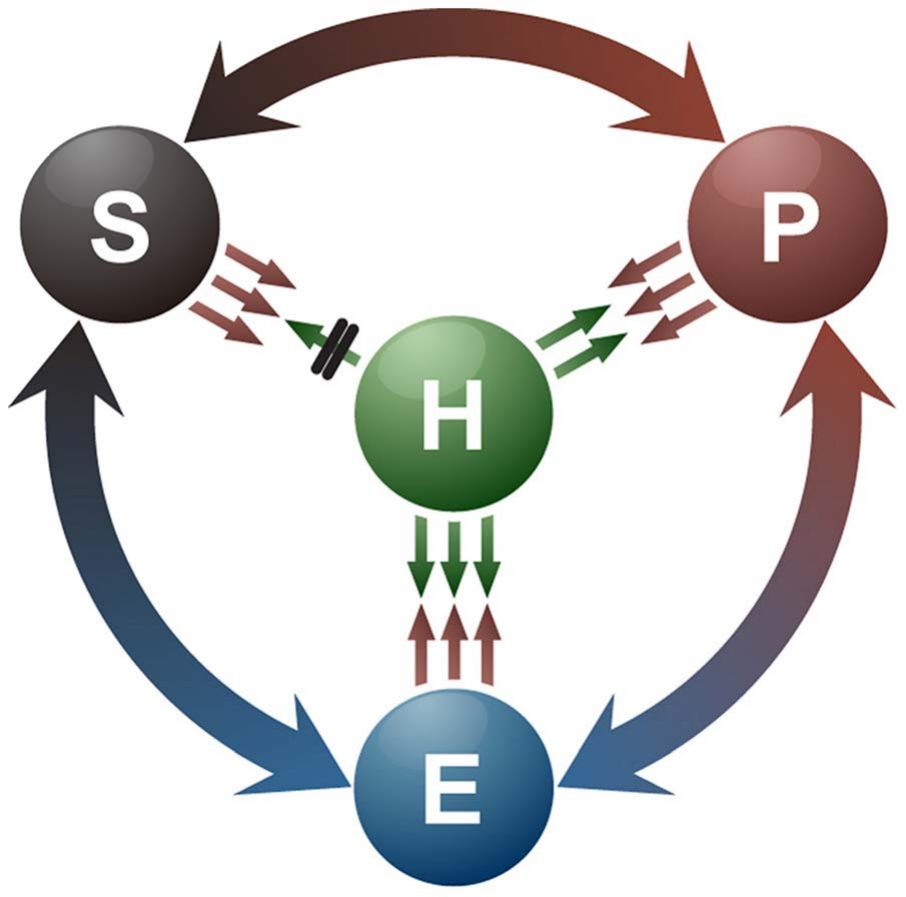
Dynamic relationship between a fungus and its host plant as a function of the fungal lifestyles, Endophyte–Pathogen–Saprotroph, as a circle (EPS Ring). The endophytic stage represents a balanced interaction between fungal virulence and host defense factors. When this balance is disturbed or the host dies, endophytes may become pathogens or saprotrophs, respectively. Saprotrophs and pathogens may switch their lifestyles to endophytes/pathogens and endophytes/saprotrophs, respectively, in the presence of appropriate environmental factors. **H**, host; **E**, endophyte; **P**, pathogen; **S**, saprotroph.

Understanding of lifestyle switching in fungi is critical for deciphering the evolution of host–microbe interactions and carbon/nitrogen cycling in the ecosystem. Our data and previous reports^8^,^9^,^10^,^11^ suggest that fungal lifestyles are not stable but dynamic, and are likely influenced by the genetic makeup of the fungal species, host factors, and changing environments. The endophytic stage represents a balanced interaction between the fungus and its host. However, endophytic fungal species can become pathogens or saprotrophs when this balance is disturbed or the host dies, respectively. In contrast, saprotrophic wood decomposers can colonize Scots pine's roots as mycorrhizal fungal species^25^. Opportunistic fungal pathogens, including *Aspergillus fumigatus*^26^,^27^ and *Candida albicans*^28^ are pathogenic only in immunocompromised humans^26^,^27^,^28^. Therefore, many fungi have likely evolved to switch their lifestyles among the **E**ndophyte–**P**athogen–**S**aprotroph as a circle (EPS Ring; Fig. 4) to adapt to various hosts and changing environmental conditions. However, the mechanisms underlying the appearance of *N. crassa* as the first fungal colonizer after forest fires in nature remain unknown. Although ascospores rather than conidia in the soil or on the tree were proposed as a source of *Neurospora* after a forest fire^6^,^29^, this is contradicted by the fact that conidia are observed most frequently in the field^5^. It has also been reported that desiccated conidia can survive after treatment at >100°C^30^ and our data also provides the evidence that *N. crassa* can survive on wood trunks after heat-treated (Fig. 2d). However, we do not rule out the possibility that ascospores could also survive such extreme temperature. These further suggest that *N. crassa* within host cells can survive and grow out from the burned tree in which it resides as a pathogen or an endophyte. To better understand the ecology of *N. crassa* and provide more ecological relevance of our findings, we attempted to detect *N. crassa* from forest soil samples collected from tropical (Indonesia) to Nordic (Finland) areas including post-forest fire sites (Supplementary Table S1). We were unable to amplify *N. crassa* ITS region by PCR from these soils. Even when soil DNA samples from post-forest fire sites were analyzed by pyrosequencing, no sequence signature was found as *Neurospora* species. These results would support our hypothesis that *N. crassa* may not be living as a saprotroph in forest soils but as an endophyte or a pathogen in their natural host, Scots pine. Taken together, our study will provide a new paradigm to understand fungal lifestyles in nature and coevolution with its associated plants.

## Methods

### Plant and fungal materials

*Neurospora crassa* strains (Supplementary Table S2) used in this study were obtained from the Fungal Genetic Stock Center, Missouri, USA, and grown on Vogel's medium (pH 6.5)^2^. *Heterobasidion* *annosum* P-type (isolate 03012 provided by Kari Korhonen, METLA, Finland) was maintained and grown on MEG medium (0.5% malt extract, 0.5% glucose, 2% agar). Scots pine (*Pinus sylvestris* L.) seeds were obtained from Svenska Skogsplantor (Saleby FP-45), Finland and sown on 2% water agar plates (or test tubes) or a mixture of sterilized Kekkilä White 420-W peat for microcosm assay. Prior to use, 100 seeds were sterilized by soaking in 30 ml of 33% Hydrogen peroxide for 15 min, and then washing with 2 L sterilized water^31^. Experiments on water agar or in microcosm were performed in growth chamber or in plant growth room, respectively. The controlled conditions were a 12-h light/12-h dark photoperiod at 24°C with 80% RH in growth chamber and a 16-h light/8-h dark period at 18°C in plant growth room.

### Infection experiments and sample preparations

Five microliters of conidial suspension (10^5^ conidia per mL^−1^ in water) were placed near the base of stems (in test tubes) or on roots (on Petri dish) of 3-week-old Scots pine seedlings on water agar. For microcosm assay, six Scots pine seeds were sown in each cup and 100 μl conidial suspensions were then placed on the peat near the roots. The plants were maintained in the plant growth room. The fungal colonization patterns were monitored over a period of 5 weeks and 5 months for the samples on water agar and in microcosm, respectively, by fluorescence and confocal microscopy. To make fungal infections in fully developed trees, 3-year-old Scots pines were used. The trees were obtained from the field in Ruotsinkylä (field station of the Finnish Forest Research Institute, Finland) and grown in natural peat (Kekkilä Oy, Finland) in a greenhouse (22°C during the day and 18°C at night). The average height and diameter of the trees were 60 cm and 15 mm, respectively. The wood dowels (autoclaved Scots pine wood, 1 cm height × 1 cm diameter) pre-colonized (4 weeks) by *N. crassa* and *H. annosum* were used as inocula. Disease symptom was measured at 6 weeks after inoculation from 9 trees for each experimental group.

For cytotoxicity assay of culture filtrate, *N. crassa* was grown in Vogel's liquid medium for 5 days and filtered with 0.22 μm filter to remove fungal mycelia and spores. Prior to use, three-week-old Scots pine seedlings were exposed to light for 30 min.

Microscopic lesions and fungal hyphae were visualized by staining infected plant tissues. The tissues were cleared in alcoholic lactophenol, consisting of 1 volume of lactophenol (phenol: glycerol: lactic acid: water, 1:1:1:1, v/v) and 2 volumes of ethanol, followed by rinsing with lactophenol. The tissues were then transferred into diluted trypan blue solution (250 μg mL^−1^ trypan blue in lactophenol) and boiled for 1 min. Destaining was performed by replacing the staining solution with chloral hydrate (250 g/100 ml), followed by a wash in 50% glycerol; and tissues were then mounted on glass slides for microscopic observation. Cell death was detected by staining with Evans blue solution (0.25% [w/v] in 0.1 mM CaCl_2_, pH 5.6; Sigma)^17^.

Diaminobenzidine (DAB) was used to detect accumulated H_2_O_2_^32^. Aniline blue staining was used to reveal callose formation^33^. Propidium iodide (PI)^34^,^35^ and wheat germ agglutinin (WGA) from *Triticum vulgaris* conjugated to fluorescein isothiocyanate (FITC) were used to stain plant and fungal cell walls, respectively.

### Wide-field fluorescence microscopy and light microscopy

Wide-field fluorescence microscopy was performed using a Leitz Laborlux S with a Leitz NPL Fluotar 100×/1.32 oil 160/0.17 objective. A fluorescein filter set with excitation at 395 nm and emission at 495 nm was used. Light microscopy (Olympus CX31) was performed with a PlanC N 10×/0.25 or a PlanC N 40×/0.65 objective.

### Confocal laser scanning microscopy

Confocal laser scanning microscopy was performed using a Leica TCS SP5II HCS A inverted microscope with an HCX PL APO 20× (0.7 NA) CS (air) objective and an HCX PL APO 63× (1.2 NA) W Corr/0.17 CS (water) objective. GFP-, FITC- and FM4-64-labeled images were captured simultaneously using 488 nm excitation with an argon laser and fluorescence detection at 500–520 nm (for GFP and FITC) and >600 nm for FM4-64^36^. The fluorescence excitation maximum for PI was 535 nm and the emission maximum was 617 nm. Image analysis was performed using Imaris ver. 7.5.2 (Bitplane Scientific Software, Zurich).

### Imaging by scanning electron microscope

Specimens were dissected into 0.5 mm with a scalpel, dried on the sterilized and uncoated cellophane placed on water solid medium for 24 h at room temperature. The cellophane with specimens was cut into 1 cm- by-1 cm square and fixed with fixing solution (2% glutaraldehyde, 2% formaldehyde, 0.1% tannic acid, 4.5% sucrose in 70 mM sodium phosphate buffer (pH 7.4) and immersed overnight at 4°C. Specimens were subsequently dehydrated with serial concentrations of ethanol (50–100%), critical point drying using Bal-Tec CPD 030 device, mounted to aluminum specimen stubs, and coated with platinum using Platinum sputter for 20 sec. The specimen was examined at 5 kV beam voltage, spot size 4.5, and 60 Pascal pressure. Digital images were captured by FEI Quanta 250 Field Emission Gun Scanning Electron Microscope (FEI Co., Eindhoven, The Netherlands) using a Large Field Low vacuum secondary electron detector (LFD).

### RNA isolation, cDNA synthesis, and qRT-PCR

RNA extraction from inoculated Scots pine seedlings was performed using TRIzol reagent (Invitrogen) according to the manufacturer's protocol. There are two sets of control, RNAs from uninoculated plants and *Neurospora crassa* grown in the culture medium. Reverse transcription was performed with Moloney murine leukemia virus reverse transcriptase (M-MuLV RT) (Thermo Fisher Scientific). qRT-PCR was performed using a LightCycler® 480 Instrument II with SYBR Green I Master (Roche). The thermal profile for qRT-PCR (primers listed in Supplementary Tables S3 and 4) was an initial 95°C for 5 min, denaturation at 94°C for 10 sec (4.8°C/s), annealing at 59°C for 10 sec (2.5°C/s), extension at 72°C for 10 sec (4.8°C/s), 40 cycles of amplification, and a final extension at 72°C for 3 min. Ct values were calculated using the LightCycler 480 software.á-tubulin (*TUBA*) and 40 S ribosome were used as reference genes to normalize the data for the host plant and the fungus, respectively. The relative fold changes were calculated by the equation (1) 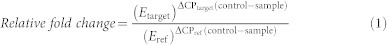


### DNA extraction and PCR from forest soil samples

A total of 115 forest soil samples collected from tropical (Indonesia) to Nordic areas (Finland) including post-forest fire sites were obtained from the courtesy of Kajar Koster, Department of Forest Sciences, University of Helsinki. Details on collection sites and year, and soil types are described in Supplementary Table S1. Soil DNA was extracted by using cetyltrimethyl ammonium bromide (CTAB) buffer^37^, and used to detect *N. crassa* by PCR with ITS primer pair of NcITS-F (AAAACTCCCACAAACCATCG) and NcITS-R (CCGCCACTGATTTTGAGG).

## Author Contributions

Conceived and designed the experiments: H.C.K., Y.H.L. Performed the experiments: H.C.K. Analyzed the data: H.C.K., S.H., J.C., F.O.A., J.P.T.V., Y.H.L. Wrote the paper: H.C.K., Y.H.L.

## Supplementary Material

Supplementary Information Supplementary information-Secret lifestyles of Neurospora crassa

## Notes

**Additional information**: Reprints and permissions information is available at www.nature. com/reprints.
