# Supplementary material for: Secret lifestyles of Neurospora crassa
Source: Sci Rep. 2014 May 30;4:5135. doi: 10.1038/srep05135 (PMC4038807; doi:10.1038/srep05135)
Supplement: Supplementary Information — Secret lifestyles of Neurospora crassa [file srep05135-s1.pdf]

Supplementary information

Secret lifestyles of *Neurospora crassa*

Hsiao-Che Kuo, Sun Hui, Jaeyoung Choi, Frederick O. Asiegbu, Jari P. T. Valkonen,

Yong-Hwan Lee

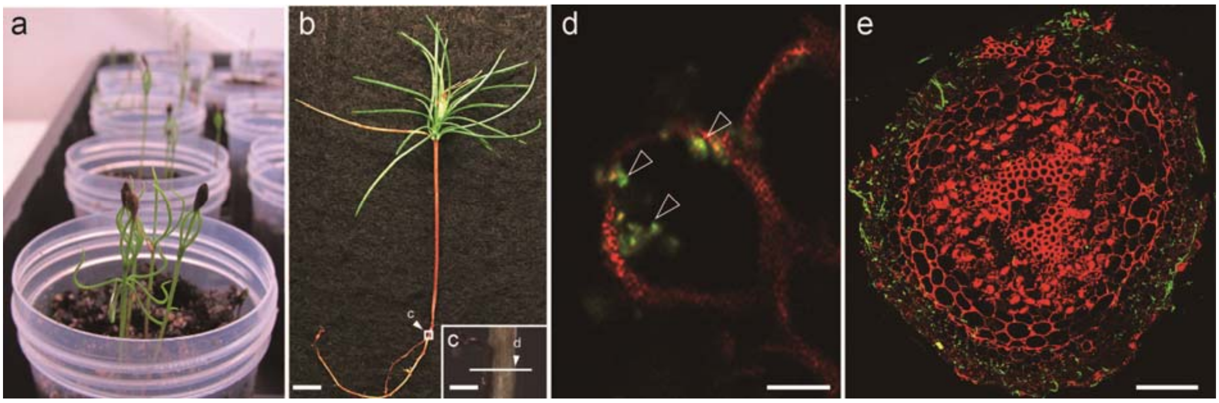

Supplementary Figure S1. Endophytic lifesyle of *N. crassa* in Scots pine. (**a**) Scots pine seedlings grown in microcosm for 2 weeks before inoculation. (**b**) Scots pine seedling 5 months post inoculation (mpi) by *N. crassa* FGSC 10589 in microcosm. (**c**) Area of sampling for microscopic observation. (**d**) *N. crassa* hyphae expressing GFP (arrows) in the root of Scots pine seedling at 5 mpi. (**e**), Transverse section of Scots pine root inoculated by *N. crassa* FGSC 2489 on water agar. Seedling survived healthy without any symptom until 3 mpi. Plant cell walls were stained with PI, and fungal hyphae were labeled with WGA. Bars = 1 cm (**b**); 1 mm (**c**); 5 µm (**d**); 100 µm (**e**).

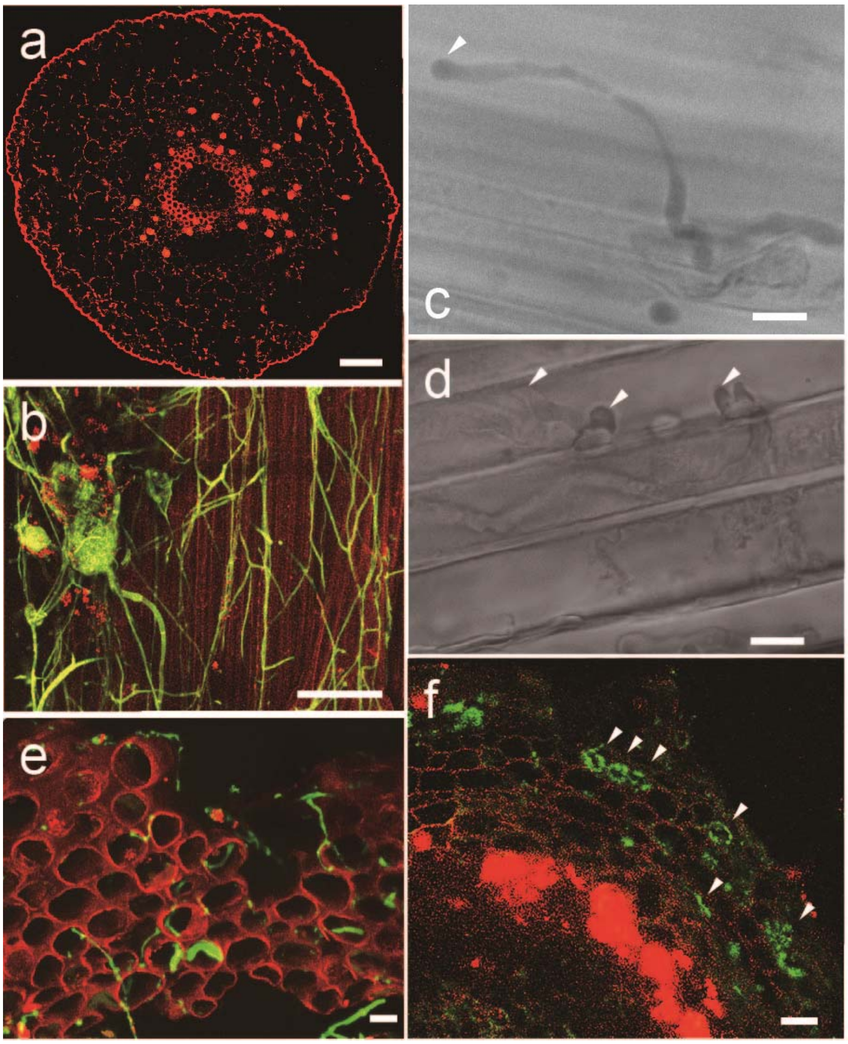

Supplementary Figure S2. Association of *N. crassa* with Scots pine. (**a**) Transverse section of 3-week-old uninfected seedling stained with PI. (**b**) *N. crassa* hyphae (stained with WGA) grown on the root surface at 3 wpi. (**c**) A hyphopodia-like structure (white arrowhead) on the root surface. Fungal hyphae were stained with trypan blue. (**d**) *N. crassa* hyphae (white arrowheads) penetrate and grow within plant tissue (stem). (**e**) Three dimensional (3D) confocal image of Scots pine's root tissue at 3 wpi with *N. crassa*. (**f**) Invasive growth of *N. crassa* FGSC 10589 from one cell to an adjacent cell at 3 wpi (GFP of fungal hyphae are indicated by white

arrowheads). Bars = 100 µm (**a**, **b**, and **f**); 10 µm (**c**, **d**, and **e**). *N. crassa* strain used in a, b, c, d, and e was FGSC 2489.

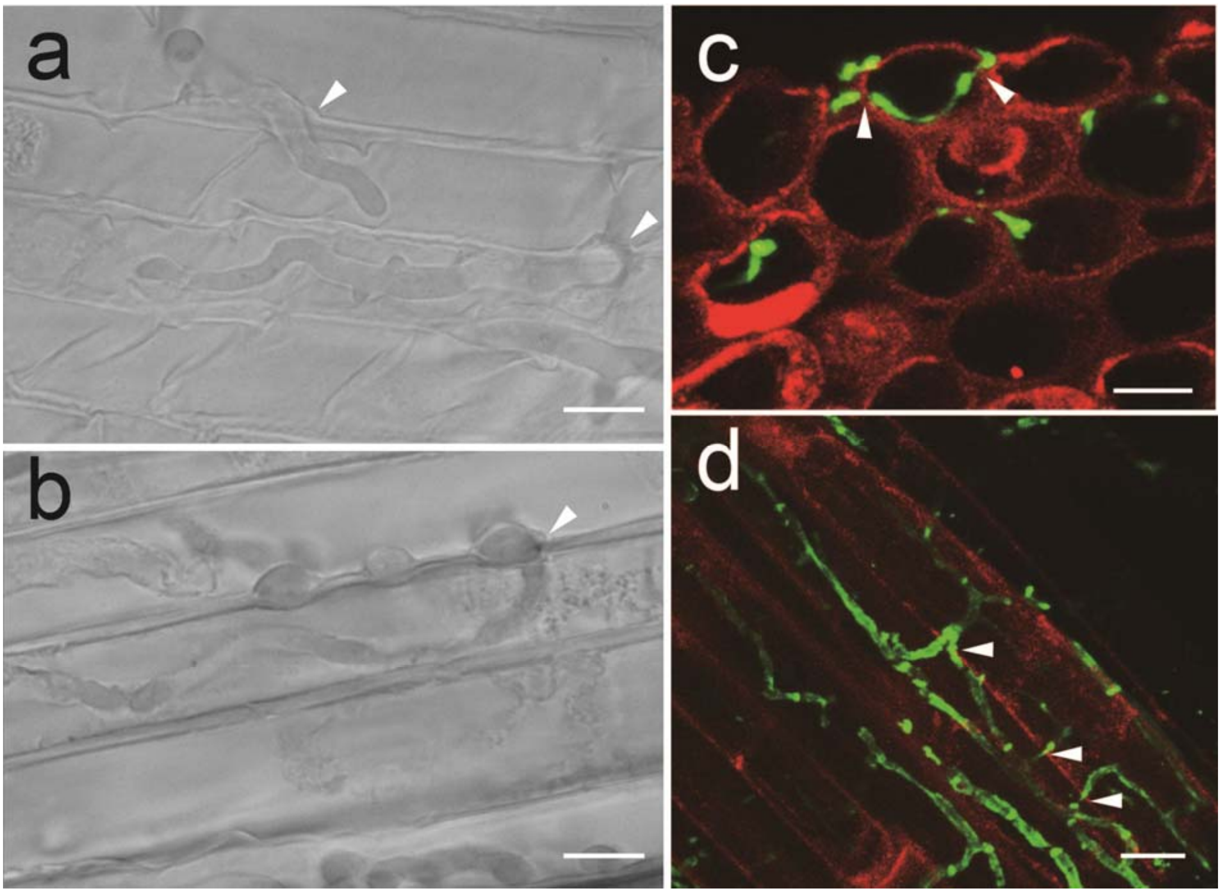

Supplementary Figure S3. Intercellular growth of *N. crassa* in Scots pine cells. (**a and b**) *N. crassa* hyphae penetrate and grow within plant tissue (stem). (**c**) Three dimensional (3D) confocal image of Scots pine's root tissue at 3 wpi with *N. crassa*. (**d**) Image of *N. crassa* hyphae stained with WGA within host plant cells. White arrowheads indicate the places where *N. crassa* hyphae penetrate through cell wall. Bars = 10 μm. *N. crassa* strain used in a, b, c, d was strain FGSC 2489.

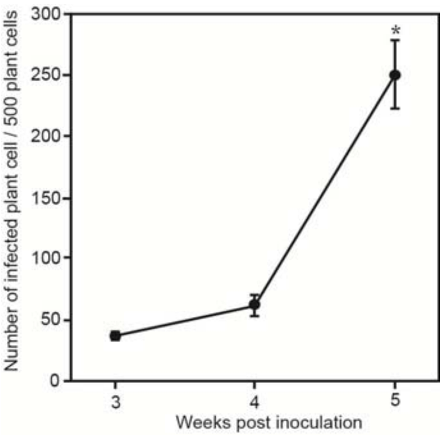

Supplementary Figure S4. Infection progress of *N. crassa* in Scots pine's roots. The number of infected cells by *N. crassa* was counted in Scots pine's roots from 3 to 5 wpi. There were 3 biological replicates (500 cells from 10 trees each) at each time point. The bars indicate standard deviation among biological replicates. The number of infected cells was significantly increased in 5 wpi compared to 3 and 4 wpi. *, $p < 0.01$.

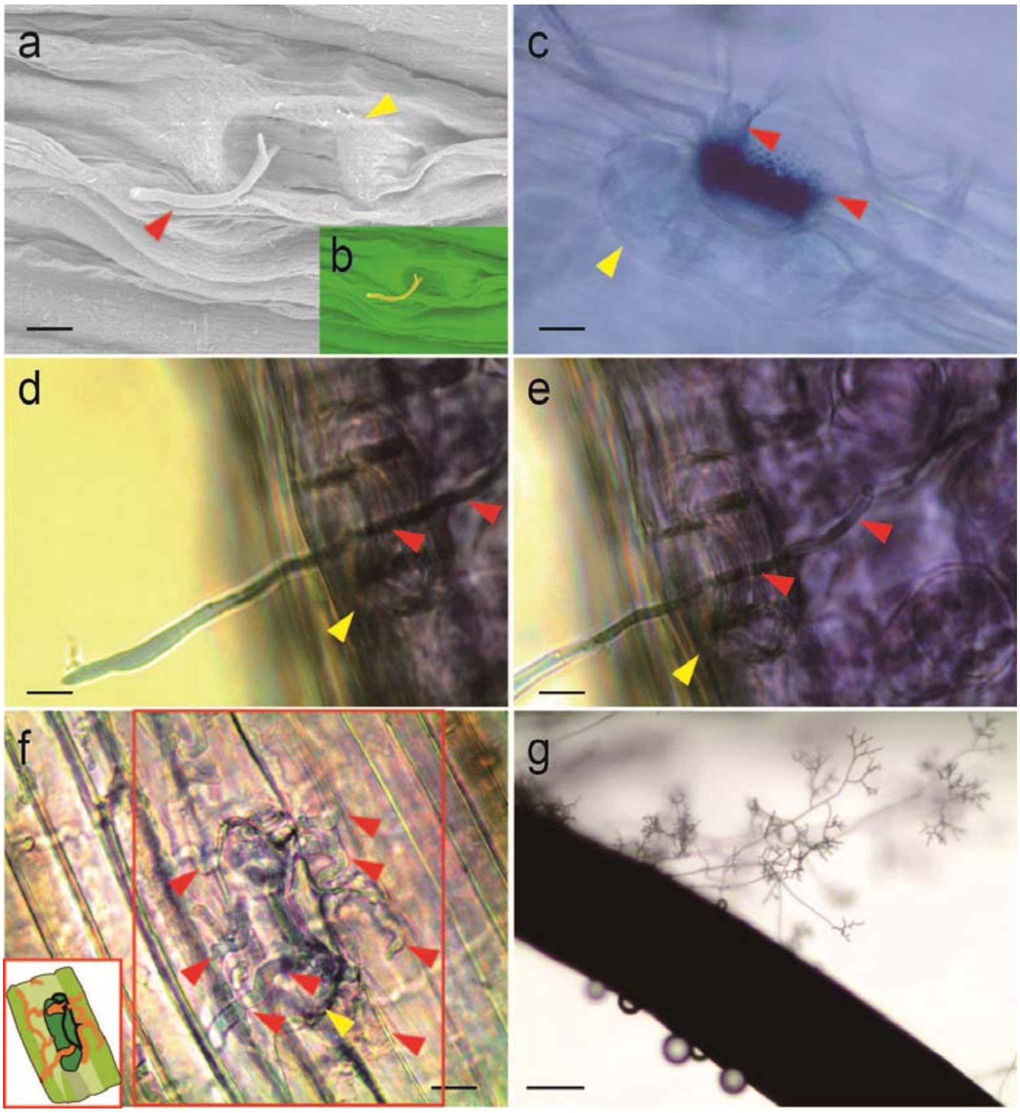

Supplementary Figure S5. Asexual reproduction of *N. crassa* on a living Scots pine. (**a–f**) *N. crassa* hyphae growing out from stomata of the stems of Scots pine seedlings at 3 wpi. (**c**) sample strained with trypan blue. (**d**) hyphal tip. (**e**) hyphae inside host plant. (**g**) *N. crassa* growing out from an infected pine seedling formed conidiophores with conidia (asexual spores) on needles. Bars = 10 µm (**a–f**); 500 µm (**g**). Yellow and red arrowheads indicate stomata and *N. crassa* hyphae, respectively.

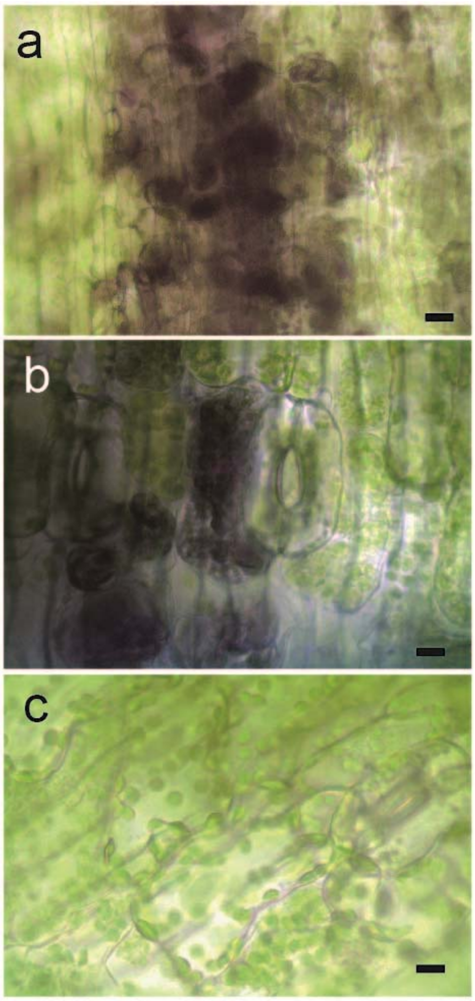

Supplementary Figure S6. Host cell death by infection and culture filtrate of *N. crassa*. (**a**) Cell death caused by inoculation of *N. crassa*. (**b**) Cell death caused by culture filtrate of *N. crassa*. (**c**) Control treated by culture medium. Dead plant cells were visualized by staining with Evan blue.

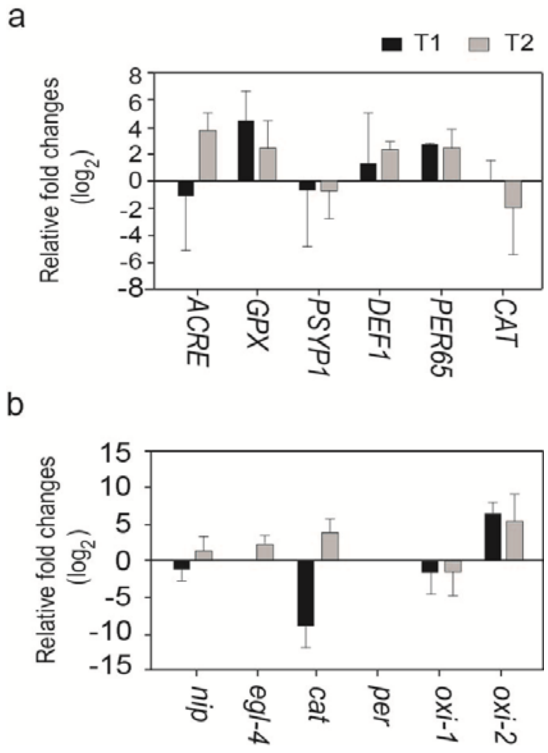

Supplementary Figure S7. Gene expression patterns during interactions between Scots pine and *N. crassa*. (**a**) Expression patterns of defense-related genes in Scots pine's stem after *N. crassa* infection were measured at one (T1) and two (T2) weeks after inoculation. *ACRE*, Avr9/Cf-9 rapidly elicited defense-related gene; *DEF1*, defensin; *PER65*, peroxidase 65; *PSYP1*, Class III peroxidase; *GPX*, glutathione peroxidase; and *CAT*, catalase. (**b**) Expression patterns of *N. crassa* genes were measured at one (T1) and two (T2) weeks after inoculation. *nip*, necrosis-inducing protein; *cat*, catalase-1; *per*, dyp-type peroxidase; and *oxi-1* and *oxi-2,* two oxidoreductases. RNAs from uninfected trees and *N. crassa* grown in culture medium were used as controls for defense-related and fungal genes, respectively. α-tubulin (*TUBA*) and 40S ribosome genes were used as reference genes to normalize the data for the host plant and the fungus, respectively. There were 3 biological replicates for each experiment. The bars indicate standard deviation among biological replicates.

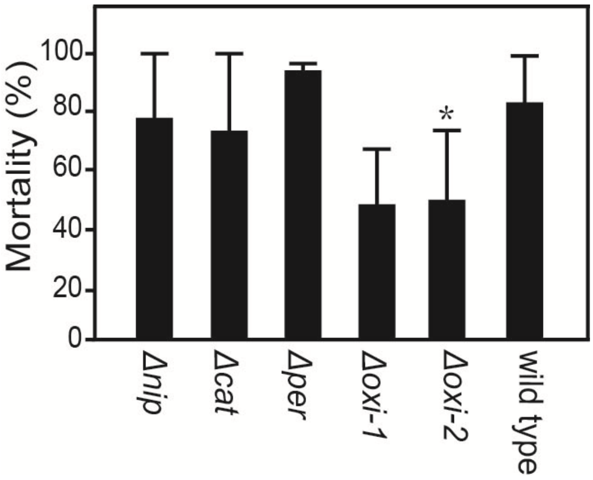

Supplementary Figure S8. Mortality of Scots pine seedlings by wild-type (FGSC 2489) and mutant strains of *N. crassa*. Inoculated plants were incubated in a growth chamber and mortality was recorded 5 wpi. *Δnip*, FGSC 13784; *Δcat*, FGSC 11258; *Δper*, FGSC 14417; *Δoxi-1*, FGSC 19367; and *Δoxi-2*, FGSC 12090. *, $p < 0.05$. There were 3 biological replicates (9 trees each). The bars indicate standard deviation among independent experiments.

Supplementary Table S1. Forest soil samples used in this study

Location and soil type	Collection date	Number of samples
Indonesia; Mineral soil	June 2010	3
Finland; Mineral soil; Post-forest fire site	August 2011	52
Finland; Peatland	June 2010	15
Finland; Peatland	June 2010	15
Finland; Mineral soil	June 2010	9
Finland; Mineral soil	March-October 2012	21

Supplementary Table S2. Fungal strains used in this study

FGSC#*	Strain information	NCU#†
2489	*Neurospora crassa* 74-OR23-1A	
10589	*Neurospora crassa* Pccg-1::sGFP::bar+(EC)	
13784	*Neurospora crassa* necrosis inducing protein mutant	02338
11258	*Neurospora crassa* catalase-1 mutant	08791
14417	*Neurospora crassa* dyp-type peroxidase mutant	09210
19367	*Neurospora crassa* oxidoredutase mutant	00520
12090	*Neurospora crassa* oxidoredutase mutant	06061
	Heterobasidion annosum	

*FGSC, Fungal Genetics Stock Center

†NCU, Locus number of annotated gene

Supplementary Table S3. qRT-PCR primers for *P. sylvestris* genes

Target gene	Name of primer	Sequence	Genbank acc./EMBL acc.
defensin (*DEF1*)	PS-Def1-RT-F	CGTGCTGCTTGTTATAACCA	EF455616
	PS-Def1-RT-R	CAACTTCCCGTTGGAAATCC	
peroxidase 65 (*PER65*)	PS-PER65-RT-F	GCCCAGAATTTATTGTTGGA	EL342386
	PS-PER65-RT-R	CTGTAAATATAAGCTCCCTGGT	
Avr9/Cf-9 rapidly elicited defense-related gene (*ACRE*)	PS-DR-RT-F	GTAAAGTCTATGGAAGAAGCCT	AY423270
	PS-DR-RT-R	TGAATGCATGGAAAGTGCAG	
class III peroxidase PSYP1 (*PSYP1*)	PS-PSYP1-RT-F	GTGATTGTATATGGTAGCGCTG	AF291667
	PS-PSYP1-RT-R	GTCTGCACTCAAATAGACTTCC	
glutathione peroxidase (*GPX*)	PS-GPx-RT-F	ATGCCAATTTAGCACCAAGG	FN546175
	PS-GPx-RT-R	GCTTTCCCATGCAATCAGTT	
catalase (*CAT*)	PS-CAT-RT-F	AAGGGCTTTTTCGAGGTGAC	FN546173
	PS-CAT-RT-R	GGAATTACCTGCATGGCATC	
α-tubulin (*TUBA*)	PS-Tub-RT-F	GATCTGGTTCAGCCTGATGG	FN546172
	PS-Tub-RT-R	CCAGTCCGTACCTCGTCAAT	

Supplementary Table S4. qRT-PCR primers for *Neurospora crassa* genes

Target gene	Name of primer	Sequence	NCU#
necrosis inducing protein (*nip*)	02338F	CTCTCGACTTTGACACCGAC	02338
	02338R	AGAGATAAATGCACCAGCCG	
endoglucanase IV (*egl-4*)	07760F	CATCGACCAGAAGGACAACG	07760
	07760R	GTCGATCTTGAAGAACTTGAGGG	
catalase-1 (*cat*)	08791F	CAGAACCACCCACAGAACAC	08791
	08791R	GAAGCGCATGATCCTTTCAC	
dyp-type peroxidase (*per*)	09210F	CTGTTCGGTACAGCAATGAC	09210
	09210R	CGAAGATCAATCCTGACTTGAC	
oxidoredutase (*oxi-1*)	00520F	CTGAAGAAATCGAAGAACACTCTC	00520
	00520R	GTGGATCTTGTAGATGTCGGTC	
oxidoredutase (*oxi-2*)	06061F	TGGCTTCTACCATCTCATTGG	06061
	06061R	CTCAAAGAGAAACTGCTTCCTG	
40S ribosome	NrDNAF	TCACATGCAGCCTTACCTC	03393
	NrDNAR	GACGATCTTCTCCCAGGTC	
